# Validation of Online Versions of Tinnitus Questionnaires Translated into Swedish

**DOI:** 10.3389/fnagi.2016.00272

**Published:** 2016-11-22

**Authors:** Karolina Müller, Niklas K. Edvall, Esma Idrizbegovic, Robert Huhn, Rilana Cima, Viktor Persson, Constanze Leineweber, Hugo Westerlund, Berthold Langguth, Winfried Schlee, Barbara Canlon, Christopher R. Cederroth

**Affiliations:** ^1^Center for Clinical Studies, University Medical Center RegensburgRegensburg, Germany; ^2^Experimental Audiology, Department of Physiology and Pharmacology, Karolinska InstitutetStockholm, Sweden; ^3^Hörsel-och Balanskliniken, Karolinska UniversitetssjukhusetStockholm, Sweden; ^4^Clinical Psychological Science, Faculty of Psychology and Neuroscience, Maastricht UniversityMaastricht, Netherlands; ^5^Center of Expertise in Rehabilitation and Audiology, Adelante RehabilitationHoensbroek, Netherlands; ^6^Stress Research Institute, Stockholm UniversityStockholm, Sweden; ^7^Department of Psychiatry and Psychotherapy, University Medical Center RegensburgRegensburg, Germany

**Keywords:** tinnitus, questionnaires, anxiety, depression, stress, hyperacusis, quality of life, TFI

## Abstract

**Background:** Due to the lack of objective measures for assessing tinnitus, its clinical evaluation largely relies on the use of questionnaires and psychoacoustic tests. A global assessment of tinnitus burden would largely benefit from holistic approaches that not only incorporate measures of tinnitus but also take into account associated fears, emotional aspects (stress, anxiety, and depression), and quality of life. In Sweden, only a few instruments are available for assessing tinnitus, and the existing tools lack validation. Therefore, we translated a set of questionnaires into Swedish and evaluated their reliability and validity in a group of tinnitus subjects.

**Methods:** We translated the English versions of the Tinnitus Functional Index (TFI), the Fear of Tinnitus Questionnaire (FTQ), the Tinnitus Catastrophizing Scale (TCS), the Perceived Stress Questionnaire (PSQ-30), and the Tinnitus Sample Case History Questionnaire (TSCHQ) into Swedish. These translations were delivered via the internet with the already existing Swedish versions of the Tinnitus Handicap Inventory (THI), the Hospital Anxiety and Depression Scale (HADS), the Hyperacusis Questionnaire (HQ), and the World Health Organization Quality of Life questionnaire (WHOQoL-BREF). Psychometric properties were evaluated by means of internal consistency [Cronbach's alpha (α)] and test–retest reliability across a 9-week interval [Intraclass Correlation Coefficient (ICC), Cohen's kappa] in order to establish construct as well as clinical validity using a sample of 260 subjects from a population-based cohort.

**Results:** Internal consistency was acceptable for all questionnaires (α > 0.7) with the exception of the “social relationships” subscale of the WHOQoL-BREF. Test–retest reliability was generally acceptable (ICC > 0.70, Cohens kappa > 0.60) for the tinnitus-related questionnaires, except for the TFI “sense of control” subscale and 15 items of the TSCHQ. Spearmen rank correlations showed that almost all questionnaires on tinnitus are significantly related, indicating that these questionnaires measure different aspects of the same construct. The data supported good clinical validity of the tinnitus-related questionnaires.

**Conclusion:** Our results suggest that most Swedish adaptations of the questionnaires are suitable for clinical and research settings and should facilitate the assessment of treatment outcomes using a more holistic approach by including measures of tinnitus fears, emotional burden, and quality of life.

## Introduction

Tinnitus is the perception of one or more sounds despite the physical absence of such sound(s) (Chan, 2009). This condition is chronically experienced by a large portion of the population (>15%) and severely debilitating for about 1–2% of the population, affecting sleep, concentration, and productivity at work (Dobie, 2003; Heller, 2003). Tinnitus is associated with a higher risk of receiving disability pension (Friberg et al., 2012) and perceived as an enormous socioeconomic burden (Cederroth et al., 2013). In the Netherlands, tinnitus-related costs have been estimated to be € 6.8 billion per year (Maes et al., 2013). The prevalence of tinnitus is age-dependent, peaking in the seventh decade of life (Nondahl et al., 2002; Gopinath et al., 2010a,b; Shargorodsky et al., 2010; Park B. et al., 2014; Park K. H. et al., 2014). Tinnitus remains a clinical enigma because of the lack of effective treatments for stopping phantom tinnitus perception (Chan, 2009). Presently, tinnitus assessment relies on self-report questionnaires and subjective psychoacoustic measures (Langguth et al., 2007). Tinnitus heterogeneity varies in its phenotypes and may be objective (emitted by the ear itself and perceivable by an external observer) or subjective (only perceived by the patient), chronic or occasional, pulsatile or non-pulsatile, noise or tonal, constant or intermittent, and unilateral or bilateral (Langguth et al., 2013). Tinnitus may present with a high number of etiologies (e.g., noise exposure, stress, or physical trauma) and a multitude of co-morbidities (e.g., hypertension or diabetes; Langguth et al., 2013). The large variety in tinnitus profiles is thought to partly responsible for the lack of success in clinical treatment trials (Tunkel et al., 2014). Thus, tools need to be urgently identified for reliably assessing tinnitus and enabling the classification of patient subgroups according to a defined set of characteristics.

Several efforts have been made to establish a consensus for patient assessment and outcome measurement (Langguth et al., 2007; Landgrebe et al., 2012; Zeman et al., 2012, 2014). Nevertheless, a recent systematic review has shown that more than 100 instruments are used for primary outcome measures in clinical trials (Hall et al., 2016), evincing that there is still no agreement on how to assess tinnitus. For this reason, a working group of the Cooperation in Science and Technology (COST) action TINNET, a European Tinnitus research Network (www.tinnet.tinnitusresearch.net), is currently standardizing assessment methods and defining a core set of domains and instruments (Hall et al., 2015).

In Sweden, national guidelines on the management and treatment of tinnitus are lacking, and clinics in the different counties rely on local recommendations. The only questionnaires recommended are the Tinnitus Handicap Inventory (THI) and the Hospital Anxiety and Depression Scale (HADS). However, the Swedish versions of these questionnaires lack validity. Thus, the number of patients with tinnitus in Sweden receiving appropriate care is rather small when compared to the large capacities of other European clinics (Karolinska Hospital in Stockholm Sweden, *n* = 70 patients per year vs. the Tinnitus Clinic at the Charité in Berlin, *n* = 3000 new patients per year; or in the Adelante Tinnitus Expert Center in Maastricht, Netherlands, *n* = 700 newly referred patients per year), even when considering the population size of the respective cities. We selected a number of additional questionnaires (for instance, the Tinnitus Sample Case History Questionnaire (TSCHQ), Tinnitus Functional Index (TFI), Fear of Tinnitus Questionnaire (FTQ), Tinnitus Catastrophizing Scale (TCS), and Perceived Stress Questionnaire (PSQ-30) according to recommendations given in a consensus meeting (Langguth et al., 2007) or because of their successful application in clinical trials on tinnitus (Cima et al., 2012). Each of the questionnaires was translated into Swedish. A set of validated questionnaires would not only enable Swedish clinics to assess the burden of tinnitus in a wider context but also other aspects such as measures of tinnitus fears, emotional burden, and quality of life.

## Materials and methods

### Subjects

Patients with tinnitus were identified in the fifth wave of the Swedish Longitudinal Occupational Survey of Health (SLOSH). All patients aged between 18 and 85 years who had previously agreed to be contacted (*n* = 620) were invited to join STOP and participate in an online survey. Additionally, 319 participants were recruited through flyers. Two hundred and seventy one subjects registered with STOP (http://stop.ki.se) gave their written informed consent to participate in the survey. After excluding participants without tinnitus and incomplete test–retest data, a total sample size of 260 subjects was achieved. The project was approved by the local ethics committee “*Regionala etikprövningsnämnden*” in Stockholm (2014/1998-31/4). The database project and the server were coordinated and located at the Department of Physiology and Pharmacology of the Karolinska Institutet, Sweden.

### Selection of questionnaires

Based on a consensus meeting in 2006, Langguth et al. (2007) recommended the use of several questionnaires such as the TSCHQ (Landgrebe et al., 2010), the THI (Newman et al., 1996, 1998), the Tinnitus-Beeinträchtigungs-Fragebogen (TBF-12; Greimel et al., 1999), the Major Depression Inventory (MDI; Bech and Wermuth, 1998), and the World Health Organization-Quality of life questionnaire (WHO, 1998). These questionnaires have been used in a large number of studies, albeit preferentially in Europe (Hall et al., 2016).

The TSCHQ was designed to assess the most important tinnitus characteristics and the tinnitus history of patients (Landgrebe et al., 2010). Tinnitus-related impairment in daily life is typically assessed with the THI (Newman et al., 1996, 1998). The TFI (Meikle et al., 2012; Henry et al., 2016) has been proposed as a more recent questionnaire with very high internal consistency of 0.97 and test–retest reliability of 0.78. We favored the TFI over the TBF-12 because of its high responsiveness to treatment-related changes.

In a randomized controlled trial on cognitive behavioral therapy (CBT) that included 245 patients with tinnitus, Cima et al. (2012) reported the successful and valid use of various questionnaires developed for assessing tinnitus-related emotional affects. Tinnitus-specific emotional reactivity and cognitions were evaluated with the TCS and the FTQ (Cima et al., 2011). The TCS is used for assessing cognitive misinterpretations of tinnitus sounds and the FTQ for measuring tinnitus-related fears (Cima et al., 2011). Both questionnaires showed excellent internal consistency values (TCS: Cronbach's alpha = ·0.94; FTQ: Cronbach's alpha = 0.82). Moreover, Cima et al. (2011) evaluated negative emotional affects with the HADS that also showed good reliability (Cronbach's alpha = 0.71–0.90; Spinhoven et al., 1997). The HADS is used for evaluating both depression and anxiety and has been previously tested on the Swedish tinnitus population (Andersson et al., 2003). Therefore, we decided to replace the MDI recommended in the 2006 consensus meeting (Langguth et al., 2007) that only evaluates depression and used the HADS instead. Stress is widely evaluated with the PSQ-30 showing an internal consistency of 0.80 < α < 0.86 (Levenstein et al., 1993). The combination of HADS and PSQ-30 allows the distinct evaluation of stress, anxiety and depression.

No Hyperacusis Questionnaire (HQ) was suggested in the initial recommendation (Langguth et al., 2007). However, because about 40–55% of patients with tinnitus experience this condition (Baguley, 2003; Schecklmann et al., 2014), we also considered the HQ (Khalfa et al., 2002), which had been validated in a group of tinnitus patients showing an internal consistency of α = 0.88 (Fackrell et al., 2015). A Swedish version was developed with an internal consistency of α = 0.92, albeit tested on people with Williams Syndrome (Blomberg et al., 2006).

The Health Utilities Index (HUI)—validated for assessing quality of life of patients with tinnitus (Maes et al., 2011)—was used as a primary outcome measure to evaluate the efficacy of specialized CBT on quality of life (Cima et al., 2012). However, a quality of life questionnaire developed by the WHO has also been shown to be suitable for patients with tinnitus (Zeman et al., 2014). The World Health Organization Quality of Life Scale (WHOQoL-BREF), which is a shorter version of the long questionnaire (WHO, 1998), is already available in many different languages and appears to be more appropriate for world-wide use than the HUI.

Permission to translate the questionnaires into Swedish was obtained from all developers of source language questionnaires: B. Langguth and W. Schlee (TSCHQ), J. A. Henry (TFI), R. R. L. Cima (FTQ and TCS), S. Levenstein (PSQ-30). For the TFI translation, as the reproduction in whole or in part is prohibited without the written consent of Oregon Health & Science University (OHSU), a license was obtained from OHSU, who agreed on the above procedure and authorized the validation of the translated TFI questionnaire. For further use in the clinics in Sweden, additional agreements will be needed.

### Translation

No clear guidelines exist on how to translate questionnaires (Epstein et al., 2015), in particular when cultural adaptations are required as in the case of translations from English into Swedish. Since the objective of our translations was to find a functional equivalent but not a literal formulation of the original versions, we relied on a procedure called TRAPD (translation, review, adjudication, pre-testing, and documentation) developed by Harkness (2003). This procedure includes translators as well as a team reviewing the translations and presenting the final version (Harkness, 2003). The original English versions of the questionnaires were thus translated into Swedish by three native Swedish speakers (whose mother tongue was the target language, who were fluent in English and country residents with experience in the target culture). All translators were briefed on the background of the project before the translation. First, all translators worked independently and then in a team to produce one single reconciled forward translation. This forward translation was then reviewed and discussed by a multidisciplinary committee from our clinic that included a doctor, an ENT specialist, an audiologist, a psychologist, two researchers, and a statistician to provide an additional level of quality control. All members of the reviewing committee agreed on the final version. Some of the questions and responses were slightly modified in order to produce fully comprehensible items in the Swedish language. Backward translation was conducted by a blinded native Swedish and fluent English speaker, with no knowledge of the original questionnaire. The backward-translated version was evaluated by the project leader and the translator and used as a tool to ensure that the meaning of the items was not altered (conceptual accuracy), rather than as a measure of translation accuracy. The Swedish versions of the questionnaires are available upon request.

### Online survey

Before field-testing from October 2015 to January 2016, we carried out a pilot test of these online surveys on a small group of respondents (*n* = 6) in order to detect any flaws in routing, layout, comprehension, length, software use (different browsers and mobile devices), and data transfer to the server. After giving written consent, patients were invited to participate in a secure online survey that included sociodemographic variables as well as the following questionnaires: the TSCHQ (Landgrebe et al., 2010), THI (Newman et al., 1996, 1998), TFI (Henry et al., 2016), FTQ (Cima et al., 2011), TCS (Cima et al., 2011), HQ (Khalfa et al., 2002), PSQ-30 (Levenstein et al., 1993), HADS (Andersson et al., 2003), and WHOQoL-BREF (1998). Table 1 presents an overview of the questionnaires: number of items as well as total and subscale scores. Scores were based on the scoring guideline of each questionnaire. Participants had to complete the questionnaires twice. The median time interval between initial and subsequent assessment was 70 days (Q1 = 66, Q3 = 71, range = 16–94 days). We performed no interventions between the test and re-test sessions.

**Table 1:** **Questionnaire overview**.

**Questionnaire**	**Number of items**	**Total score**	**Subscale scores**	
TSCHQ	35	–	–	
THI^a^	25	Range = 0–100; categories: 1. 0–16: Negligible 2. 18–36: Light weight 3. 38–56: Moderate weight 4. 58–76: Severe weight 5. 78–100: Catastrophic	–	
TFI^a^	25	Range = 0–100; Categories: 1. 0–17: Not a problem 2. 18–31: Small problem 3. 32–53: Moderate problem 4. 54–72: Big problem 5. 73–100: Very big problem	Range = 0–100 1. Intrusive 2. Sense of control 3. Cognitive 4. Sleep	5. Auditory 6. Relaxation 7. Quality of life 8. Emotional
FTQ^a^	17	Range = 0–17	–	
TCS^a^	13	Range = 0–52	–	
HQ^a^	14	Range = 0–42; Categories: 1. ≤ 28 = No hyperacusis 2. >28 = Hyperacusis	3. Attentional (range = 0–12) 4. Emotional (range = 0–18) 5. Social (range = 0–12)	
PSQ-30^a^	30	Range = 0–1; Categories: 1. <0.34 = Low stress level 2. 0.34–0.46 = Moderate stress level 3. >0.46 = High stress level	–	
HADS^a^	14	–	Range = 0–21 1. Anxiety 2. Depression Categories: 1. 0–7 = Normal 2. 8–10 = Borderline 3. 11–21 = Abnormal	
WHOQoL-BREF^b^	26	–	Range = 4–20 1. Physical health 2. Psychological 3. Social relationships 4. Environment	Range: 1–5 single items 5. Overall quality of life 6. Overall health

*TSCHQ, Tinnitus Sample Case History Questionnaire; THI, Tinnitus Handicap Inventory; TFI, Tinnitus Functional Index; FTQ, Fear of Tinnitus Questionnaire; TCS, Tinnitus Catastrophizing Scale; HQ, Hyperacusis Questionnaire; PSQ-30, Perceived Stress Questionnaire; HADS, Hospital Anxiety and Depression Scale; WHOQoL-BREF, World Health Organization Quality of Life Scale (short version)*.

a *Higher score, higher impairment*.

b *Higher score, higher quality of life*.

### Statistical analyses

The sample and the questionnaire values underwent descriptive analysis [frequencies (*n*), percentages (%), means (*m*), standard deviations (*SD*), medians (med), and percentiles (Q1, first quartile/25th percentile; Q3, third quartile/75th percentile)]. Mann-Whitney *U*-tests were used to examine gender differences in tinnitus-related questionnaire values and Spearman's rank correlations to assess the relation between age and tinnitus-related questionnaire values.

A range of standardly-used analyses were carried out to assess the psychometric properties of tinnitus-related questionnaires. Cronbach's alpha coefficient was used to assess the internal consistency of multi-item scales based on correlations between items on the same test or subscale and to show the extent to which several items proposed to measure the same construct result in similar scores. Coefficients >0.70 are considered acceptable (Cohen, 1960; Grouven et al., 2007; see also, http://www.rehabmeasures.org/rehabweb/rhstats.aspx). Test–retest reliability is used to evaluate how stable patients respond over time. The consistency of tinnitus-related data was assessed by Cohen's kappa coefficient for categorical variables and Intraclass Correlation Coefficient (ICC) for metric variables. ICCs > 0.70 and Cohen's kappa > 0.60 are considered acceptable (Cohen, 1960; Grouven et al., 2007; see also, http://www.rehabmeasures.org/rehabweb/rhstats.aspx). Because construct validity indicates whether instruments measure the same theoretical concept, it was used to assess inter-scale correlations [Spearman's rank correlation coefficient (ρ)] within and between tinnitus-related questionnaires. Correlation coefficients ≥0.40 indicate that questionnaires or subscales measure the same aspects of tinnitus (convergent validity), whereas correlation coefficients between <0.40 indicate that questionnaires or subscales measure different aspects (discriminant validity; algebraic signs are omitted; Hays and Hayashi, 1990). Known-group comparisons were used to evaluate the clinical validity of the tinnitus-related questionnaires. The statistical significance of group differences in tinnitus occurrence, onset, and manifestation was tested with Mann-Whitney *U*-tests.

The significance level was set at *p* ≤ 0.050. The software package SPSS for Windows, Version 23, was used for all statistical analyses.

## Results

### Subject characteristics

#### Sociodemographic data

Two hundred and sixty Swedish subjects (52.3% men) were included in the study. The median age was 62.40 years (Q1 = 56.00, Q3 = 68.00, ranging from 21 to 87 years).

#### Tinnitus-related data

The majority of participants reported experiencing continuous tinnitus (86.9%). 64.6% of subjects perceived a gradual onset of tinnitus, and 65.8% described tinnitus of (very) high frequency. Most subjects reported constant tinnitus manifestation over time (73.8%) and that they had consulted with a clinician because of the condition (61.5%). The median time since the onset of tinnitus was 15.00 years (Q1 = 6.00, Q3 = 25.00, ranging from 0 to 55 years, missing information: *n* = 30). Subjects related the initial onset to the following reasons: loud blast of sound (*n* = 41, 15.8%), stress (*n* = 37, 14.2%), change in hearing (*n* = 30, 11.5%), head trauma (*n* = 2, 0.9%), whiplash (*n* = 5, 1.9%), and others (*n* = 51, 19.6%). 36.2% (*n* = 94) could not specify any specific reason. Tinnitus was reduced by music or environmental sounds in 53.5% of subjects and intensified by loud noise in 51.2% and by stress in 53.8%. Medication had no influence on tinnitus in 91.9% of subjects. On a scale from 0 to 100, the median loudness of tinnitus was 60.00 (Q1 = 35.00, Q3 = 75.00, *n* = 259), the median percentage of total awake time being aware of tinnitus was 60.00 (Q1 = 25.00, Q3 = 100.00, *n* = 225), and the median percentage of total awake time being annoyed or distressed of tinnitus was 20.00 (Q1 = 10.00, Q3 = 50.00, *n* = 226). Table 2 presents both tinnitus-related and additional clinical data (TSCHQ).

**Table 2:** **Tinnitus-related data and test–retest reliability assessed with the Tinnitus Sample Case History Questionnaire (TSCHQ)**.

	**Initial assessment**		**Test–retest reliability**
	***n***	**%**	**Cohen's kappa/Intra-class correlation coefficient**
Onset of tinnitus (exact date)	–	–	0.34
Relation of initial onset of tinnitus (etiology)			0.51
Loud blast of sound	41	15.8	
Stress	37	14.2	
Change in hearing	30	11.5	
Head trauma	2	0.8	
Whiplash	5	1.9	
Other	51	19.6	
Do not know	94	36.2	
Tinnitus occurrence			0.73
Occasionally	34	13.1	
Permanently	226	86.9	
Time of day of tinnitus emergence			0.50
When awakening	20	7.7	
In the morning	8	3.1	
Around noon	24	9.2	
In the afternoon	16	6.2	
In the evening	39	15.0	
Before sleeping	21	8.1	
Do not know	132	50.8	
Perceiving the onset of tinnitus			0.78
Gradual	168	64.6	
Abrupt	92	35.4	
Pulsation of tinnitus			0.69
Yes, with heartbeat	33	12.7	
Yes, different from heartbeat	12	4.6	
No	215	82.7	
Location of tinnitus			0.62
Right ear	25	9.6	
Left ear	35	13.5	
Both ears, worse in right ear	37	14.2	
Both ears, worse in left ear	38	14.6	
Both ears equally	79	30.4	
Inside the head	43	16.5	
Elsewhere	3	1.2	
Tinnitus manifestation over time			0.55
Intermittent	68	26.2	
Constant	192	73.8	
Loudness of tinnitus (median, Q1/Q3)	60	35/75	0.83
Tinnitus loudness variation from day to day			0.58
Yes	173	66.5	
No	87	33.5	
Percentage of total awake time of tinnitus awareness (median, Q1/Q3)	60	25/100	0.71
Percentage of total awake time being distressed by tinnitus (median, Q1/Q3)	20	10/50	0.78
Sound of tinnitus			0.56
Tone	102	39.2	
Noise	108	41.5	
Crickets	36	13.8	
Other	14	5.4	
Pitch of tinnitus			0.47
Low frequency	15	5.8	
Medium frequency	74	28.5	
High frequency	120	46.2	
Very high frequency	51	19.6	
Reduction of tinnitus by music or environmental sounds			0.44
Yes	139	53.5	
No	65	25.0	
Do not know	56	21.5	
Worsening of tinnitus by loud noise			0.54
Yes	133	51.2	
No	82	31.5	
Don't know	45	17.3	
Tinnitus affected by head movement or touch			0.66
Yes	54	20.8	
No	206	79.2	
Tinnitus affected by nap			0.60
Yes, worsening of tinnitus	7	2.7	
Yes, reducing of tinnitus	34	13.1	
No, no effect	219	84.2	
Tinnitus affected by sleep at night			0.35
Yes	49	18.8	
No	103	39.6	
Do not know	108	41.5	
Tinnitus affected by stress			0.70
Yes, worsening of tinnitus	139	53.5	
Yes, reducing of tinnitus	2	0.8	
No, no effect	119	45.8	
Tinnitus affected by medication			0.67
Yes	21	8.1	
No	239	91.9	
Contacted a clinician due to tinnitus			0.69
Yes	160	61.5	
No	100	38.5	
Number of different tinnitus treatments			0.61
0	196	75.4	
1	18	63.9	
2–4	28	10.8	
5 or more	18	6.9	
Tinnitus occurrence in family			0.74
Yes	187	71.9	
No	73	28.1	
Comorbidity			0.83
Yes	129	49.6	
No	131	50.4	
Medication			0.87
Yes	166	63.8	
No	94	36.2	
Currently under treatment for psychiatric problems			0.73
Yes	16	6.2	
No	244	93.8	
Hearing problem			0.74
Yes	220	84.6	
No	40	15.4	
Hearing aids			0.93
Yes, on both ears	74	28.5	
Yes, on the right ear	8	3.1	
Yes, on the left ear	9	3.5	
No	169	65.0	
Problems tolerating sounds			0.41
Never	41	15.8	
Rarely	44	16.9	
Sometimes	100	38.5	
Usually	49	18.8	
Always	26	10.0	
Sounds cause pain or physical discomfort			0.46
Yes	115	44.2	
No	124	47.2	
Do not know	21	8.1	
Headache			0.77
Yes	56	21.5	
No	204	78.5	
Vertigo or dizziness			0.56
Yes	72	27.7	
No	188	72.3	
Temporomandibular disorder			0.67
Yes	29	11.2	
No	231	88.8	
Neck pain			0.82
Yes	72	27.7	
No	188	72.3	
Other pain syndromes			0.65
Yes	71	27.3	
No	189	72.7	

*Test–retest reliability was assessed by Cohen's kappa coefficient for categorical variables and intra-class correlation coefficient for metric variables. ICCs > 0.70 and Cohen's kappa > 0.60 are considered acceptable. Descriptive data of Tinnitus Sample Case History Questionnaire is presented at initial assessment*.

#### Questionnaire data

The median scores and quartiles of the test and re-test sessions are presented in Table 3. At the initial assessment, the THI was 24.00 (Q1 = 14.00, Q3 = 38.00), and 10.8% of subjects described their tinnitus as *severe* to *catastrophic*. The average TFI score was 24.0 (Q1 = 14.00, Q3 = 38.00), and 16.9% of subjects described to have a big or a very big problem. Stress was evaluated by means of the PSQ (median = 0.27, Q1 = 0.16, Q3 = 0.39), and 16.2% of subjects scored high stress levels. Anxiety was measured with the HADS (median = 2.0, Q1 = 1.0, Q3 = 5.0), in which 10% of subjects showed abnormally high scores. Depression, also evaluated with the HADS (median = 4.0, Q1 = 2.0, Q3 = 8.0), showed abnormally high scores in 4.6% of subjects. The median TCS-value was 11.5 (Q1 = 5.0, Q3 = 20.0), and that of the FTQ was 4.0 (Q1 = 3.0, Q3 = 6.0); however, no subscale is available for determining severity levels. The HQ showed that 17.7% of subjects had hyperacusis according to a >28 cut-off value. Quality of life was measured with the WHOQoL subscales for physical (median = 16.0, Q1 = 13.7, Q3 = 17.7), psychological (median = 16.0, Q1 = 14.0, Q3 = 17.3), social (median = 14.7, Q1 = 13.3, Q3 = 16.0), and environmental (median = 16.5, Q1 = 15.0, Q3 = 8.0) relationships. Age was significantly associated with tinnitus-related questionnaire scores, with exception of the THI “intrusive” subscale score, the HQ “emotional” subscale score, and the TCS total score (Table 4). Correlation coefficients were small to moderate in size. In general, the older the subjects, the fewer were the impairments and the better the quality of life reported. Women tended to have more impairments and less quality of life than men (Table 4). Significant differences were found for 17 out of 25 values.

**Table 3:** **Descriptive analyses of tinnitus-related questionnaires**.

***N* = 260**		**Initial assessment**		**Subsequent assessment**	
**Questionnaire**		**med (Q1, Q3)**	**Range**	**med (Q1, Q3)**	**Range**
**THI^b^, ^c^**					
Sum score		24.0 (14.0, 38.0)	0–90	22.0 (12.0, 36.0)	0–94
		***n***	**%**	***n***	**%**
Categories	Negligible	82	31.5	97	37.5
	Light weight	110	42.3	99	38.2
	Moderate weight	40	15.4	42	16.2
	Severe weight	20	7.7	14	5.4
	Catastrophic	8	3.1	7	2.7
**TFI^b^, ^c^**					
Sum score		24.0 (14.0, 38.0)	0–90	22.0 (12.0, 36.0)	0–94
		***n***	**%**	***n***	**%**
Categories	No problem	78	30.0	84	32.4
	Small problem	57	21.9	63	24.3
	Moderate problem	81	31.2	69	26.6
	Big problem	34	13.1	28	10.8
	Very big problem	10	3.8	15	5.8
Subscales	Intrusive	43.3 (23.3, 63.3)	0–100	43.3 (23.3, 60.0)	0–100
	Sense of control	43.3 (16.7, 66.7)	0–100	43.3 (20.0, 66.7)	0–100
	Cognitive	20.0 (6.7, 43.3)	0–93	16.7 (3.3, 40.0)	0–100
	Sleep	13.3 (0.0, 43.3)	0–100	13.3 (0.0, 40.0)	0–100
	Auditory	43.3 (16.7, 69.2)	0–100	36.7 (20.0, 66.7)	0–100
	Relaxation	23.3 (10.0, 50.0)	0–100	20.0 (6.7, 50.0)	0–100
	Quality of life	20.0 (2.5, 42.5)	0–93	17.5 (2.5, 40.0)	0–100
	Emotional	6.7 (0.0, 16.7)	0–67	6.7 (0.0, 16.7)	0–67
**FTQ^b^**					
Sum score		4.0 (3.0, 6.0)	1–14	4.0 (3.0, 6.0)	1–17
**TCS^b^, ^c^**					
Sum score		11.5 (5.0, 20.0)	0–45	10.0 (4.0, 19.0)	0–52
**PSQ-30^b^, ^c^**					
PSQ index		0.27 (0.16, 0.39)	0.00–0.82	0.24 (0.13–0.39)	0.00–0.81
		***n***	**%**	***n***	**%**
Categories	Low stress level	163	62.7	171	65.8
	Moderate stress level	55	21.2	45	17.3
	High stress level	42	16.2	43	16.5
**HQ^b^, ^c^**					
Sum score		17.0 (10.0, 25.0)	0–39	17.0 (10.0, 25.0)	0–39
		***n***	**%**	***n***	**%**
Categories	No hyperacusis	214	82.3	214	82.3
	Hyperacusis	46	17.7	46	17.7
Subscales	Attentional	5.0 (2.0, 7.0)	0–12	5.0 (2.0, 7.0)	0–12
	Emotional	7.0 (4.0, 11.0)	0–18	7.0 (4.0, 77.0)	0–18
	Social	5.0 (2.0, 8.0)	0–12	5.0 (2.0, 8.0)	0–12
**HADS^b^**					
Subscales	Anxiety	4.0 (2.0, 8.0)	0–17	4.0 (2.0, 7.0)	0–18
	Depression	2.0 (1.0, 5.0)	0–15	2.0 (1.0, 5.0)	0–18
		***n***	**%**	***n***	**%**
**THI^b^, ^c^**					
Sum score		24.0 (14.0, 38.0)	0–90	22.0 (12.0, 36.0)	0–94
		***n***	**%**	***n***	**%**
Categories anxiety	Normal	193	74.2	199	76.5
	Borderline	41	15.8	37	14.2
	Abnormal	26	10.0	24	9.2
Categories depression	Normal	227	87.3	226	86.9
	Borderline	21	8.1	23	8.8
	Abnormal	12	4.6	11	4.2
**WHOQoL-BREF^a^**					
Subscales	Physical health^c^	16.0 (13.7, 17.7)	5–20	16.0 (13.7, 17.7)	6–20
	Psychological	16.0 (14.0, 17.3)	7–20	16.0 (14.0, 17.3)	7–20
	Social relationships	14.7 (13.3, 16.0)	5–20	14.7 (13.3, 16.0)	5–20
	Environment	16.5 (15.0, 18.0)	10–20	16.5 (15.0, 18.0)	10–20
	Overall quality of life	4.0 (4.0, 5.0)	1–5	4.0 (4.0, 5.0)	1–5
	Overall health	4.0 (3.0, 4.0)	1–5	4.0 (3.0, 4.0)	1–5

*THI, Tinnitus Handicap Inventory; TFI, Tinnitus Functional Index; FTQ, Fear of Tinnitus Questionnaire; TCS, Tinnitus Catastrophizing Scale; PSQ-30, Perceived Stress Questionnaire; HQ, Hyperacusis Questionnaire; HADS, Hospital Anxiety and Depression Scale; WHOQoL-BREF, World Health Organization Quality of Life Scale (short version)*.

a *Higher score, higher quality of life*.

b *Higher score, higher impairment*.

c *n = 259 at subsequent assessment*.

**Table 4:** **Internal consistency and test–retest reliability of tinnitus-related questionnaires and relations with age and sex**.

***N* = 260**	**Internal consistency**	**Test–retest reliability**		**Age**	**Sex**		
**Questionnaire**	**Cronbach's α**	**ICC (95% CI)**	***F* (*df*)**	**Spearmen's rank correlation**	**Men med (Q1, Q3)**	**Women med (Q1, Q3)**	***U*-test**
**THI total^b^, ^c^**	**0.93**	**0.90 (0.87, 0.92)**	18.665 (258)^**^	−0.26^**^	**22.0 (12.0, 37.5)**	**26.0 (16.0, 39.5)**	7165.0^*^
**TFI total^b^, ^c^**	**0.97**	**0.88 (0.85, 0.90)**	15.314 (258)^**^	−0.22^**^	**24.6 (12.4, 40.0)**	**34.8 (14.9, 20.1)**	7066.0^*^
Intrusive	0.84	0.82 (0.78, 0.86)	10.097 (258)^**^	−0.09	41.7 (23.3, 63.3)	43.3 (24.2, 63.3)	7912.5
Sense of control	0.81	0.68 (0.61, 0.74)	5.256 (258)^**^	−0.12^*^	43.3 (16.7, 66.7)	41.7 (16.7, 65.8)	8322.0
Cognitive	0.95	0.81 (0.76, 0.85)	9.494 (258)^**^	−0.23^**^	16.7 (6.7, 36.7)	25.0 (6.7, 55.8)	7003.0^*^
Sleep	0.95	0.79 (0.74, 0.83)	8.510 (258)^**^	−0.13^*^	10.0 (0.0, 35.8)	20 (0.0, 50.0)	7000.0^*^
Auditory	0.95	0.76 (0.70, 0.81)	7.255 (258)^**^	−0.15^*^	33.3 (16.7, 63.3)	46.7 (20.0, 75.8)	7451.0
Relaxation	0.94	0.79 (0.74, 0.83)	8.493 (258)^**^	−0.24^**^	20.0 (6.7, 40.0)	30.0 (10.0, 60.0)	6962.5^*^
Quality of life	0.91	0.87 (0.84, 0.90)	14.569 (258)^**^	−0.21^**^	12.5 (2.5, 35.0)	23.8 (5.0, 46.9)	6919.0^*^
Emotional	0.88	0.81 (0.77, 0.85)	9.581 (258)^**^	−0.24^**^	3.3 (0.0, 13.3)	6.7 (0.0, 20.0)	7534.0
**FTQ total^b^**	**0.71**	**0.74 (0.68, 0.79)**	6.638 (259)^**^	−0.23^**^	**4.0 (3.0, 6.0)**	**4.0 (3.0, 6.0)**	**8136.0**
**TCS total^b^, ^c^**	**0.93**	**0.82 (0.77, 0.86)**	10.315 (258)^**^	**−0.07**	**11.0 (5.0, 19.0)**	**13.0 (5.0, 21.8)**	**7857.5**
**PSQ-30 total^b^, ^c^**	**0.94**	**0.88 (0.85, 0.91)**	16.624 (258)^**^	−0.41^**^	**23 (0.13, .34)**	**0.3 (0.2, 0.4)**	6358.5^**^
**HQ total^b^, ^c^**	**0.90**	**0.88 (0.85, 0.91)**	16.141 (258)^**^	−0.18^*^	**13.0 (8.0, 20.0)**	**22.0 (14.0, 28.8)**	5165.5^**^
Attentional	0.75	0.73 (0.67, 0.78)	6.388 (258)^**^	−0.22^**^	4.0 (1.0, 6.0)	6.0 (3.0, 9.0)	5337.0^**^
Emotional	0.83	0.86 (0.82, 0.89)	13.199 (258)^**^	−0.11	6.0 (3.3, 10.0)	9.0 (5.0, 13.0)	6313.5^**^
Social	0.82	0.83 (0.78, 0.86)	10.426 (258)^**^	−0.18^*^	4.0 (2.0, 6.0)	7.0 (4.0, 9.0)	5046.5^**^
**HADS^b^**							
Anxiety	0.85	0.85 (0.81, 0.88)	12.278 (259)^**^	−0.21^**^	4.0 (2.0, 6.0)	5.0 (3.0, 9.0)	6860.5^*^
Depression	0.83	0.85 (0.81, 0.88)	12.505 (259)^**^	−0.32^**^	2.0 (1.0, 4.8)	2.5 (1.0, 6.0)	7457.0
**WHOQoL-BREF^a^**							
Physical health^c^	0.84	0.88 (0.85, 0.91)	15.628 (258)^**^	0.18^*^	16.0 (14.1, 17.7)	15.4 (12.6, 17.1)	6900.5^*^
Psychological	0.84	0.87 (0.84, 0.90)	14.721 (259)^**^	0.27^**^	16.7 (14.7, 17.3)	15.3 (13.3, 16.7)	6261.0^**^
Social relationships	0.69^d^	0.78 (0.73, 0.83)	8.192 (259)^**^	0.21^**^	14.7 (12.3, 16.0)	14.7 (13.3, 16.0)	8414.5
Environment	0.78	0.80 (0.75, 0.84)	9.020 (259)^**^	0.26^**^	16.5 (15.5, 18.0)	16.0 (15.0, 17.5)	6733.0^*^
Overall quality of life	–	0.74 (0.69, 0.80)	6.876 (259)^**^	0.25^**^	4.0 (4.0, 5.0)	4.0 (4.0, 5.0)	7091.0^*^
Overall health	–	0.71 (0.65, 0.77)	5.920 (259)^**^	0.21^**^	4.0 (3.0, 4.0)	3.0 (2.0, 4.0)	6849.5^*^

*THI, Tinnitus Handicap Inventory; TFI, Tinnitus Functional Index; FTQ, Fear of Tinnitus Questionnaire; TCS, Tinnitus Catastrophizing Scale; PSQ-30, Perceived Stress Questionnaire; HQ, Hyperacusis Questionnaire; HADS, Hospital Anxiety and Depression Scale; WHOQoL-BREF, World Health Organization Quality of Life Scale (short version)*.

** *Correlation is significant at the 0.001 level*.

* *Correlation is significant at the 0.05 level*.

a *higher score, higher quality of life*.

b *higher score, higher impairment*.

c *n = 259 at subsequent assessment*.

d *Internal consistency of domain social relationship (WHOQoL-BREF) increases to α = 0.73 without item number 21. However, the item-total correlation between item 21 and its subscale is r = 0.42, which is acceptable*.

### Psychometric properties

#### Internal consistency

Cronbach's alpha for multi-item scales ranged from 0.69 to 0.97 (see Table 4). Thus, internal consistency was acceptable, except for the WHOQoL-BREF subscale “social relationships” that fell short of reaching the conventional cut-off value of α ≤ 0.70.

#### Test–retest reliability

ICC ranged between 0.68 and 0.90 (Table 4) and Cohen's kappa from 0.34 to 0.93 (Table 2). Test–retest reliability was acceptable, except for the subscale “sense of control” of the TFI and for 15 items of the TSCHQ. Critical items of the TSCHQ were: 3b (the time of day tinnitus starts), 5 (initial onset), 7 (etiology), 10 (intermittent or constant), 11 (varying loudness), 14 (sound of tinnitus), 15 (pitch of tinnitus), 18 (different treatments), 19 (reduction by ambient sounds), 20 (tinnitus worse by noise), 22 (nap effects), 23 (tinnitus affected by night sleep), 28 (problems tolerating sounds), 29 (pain induced by noise), and 31 (vertigo or dizziness).

#### Construct validity

Spearmen's rank correlations showed that almost all tinnitus-related questionnaires were significantly related (mainly *p* < 0.001), with the exception of the correlation between the “auditory” subscale of the TFI and the “social relationships” subscale of WHOQoL-BREF (ρ = −0.12, *p* = 0.053). 55% (*n* = 165) of 300 correlations yielded coefficients of ≥0.40, and 10.3% (*n* = 31) substantial coefficients of ≥0.70. These findings indicated that the questionnaires measured different aspects of the same construct. In general, higher correlation coefficients were observed between total and subscale scores of the THI, TFI, HQ, and HADS. WHOQoL-BREF showed correlation coefficients of ≥0.40 mainly within its subscales but not with other tinnitus-related questionnaires. Table 5 summarizes the correlation coefficients.

**Table 5:** **Convergent validity: spearmen's rank correlations between tinnitus-related questionnaires**.

	**THI total**	**TFI total**	**TFI intrusive**	**TFI sense of control**	**TFI cognitive**	**TFI sleep**	**TFI auditory**	**TFI relaxation**	**TFI quality of life**	**TFI emotional**	**FTQ total**	**TCS total**	**HQ total**	**HQ attentional**	**HQ emotional**	**HQ social**	**PSQ-30 total**	**HADS anxiety**	**HADS depression**	**WHOQoL physical health**	**WHOQoL psychological**	**WHOQoL social relationships**	**WHOQoL environment**	**WHOQoL overall quality of life**	**WHOQoL overall health**
THI total	–																								
TFI total	0.85^**^	–																							
TFI intrusive	0.70^**^	0.85^**^	–																						
TFI sense of control	0.67^**^	0.77^**^	0.68^**^	–																					
TFI cognitive	0.78^**^	0.89^**^	0.71^**^	0.66^**^	–																				
TFI sleep	0.60^**^	0.73^**^	0.58^**^	0.48^**^	0.67^**^	–																			
TFI auditory	0.61^**^	0.73^**^	0.61^**^	0.45^**^	0.55^**^	0.39^**^	–																		
TFI relaxation	0.71^**^	0.86^**^	0.71^**^	0.65^**^	0.81^**^	0.67^**^	0.48^**^	–																	
TFI quality of life	0.79^**^	0.89^**^	0.66^**^	0.59^**^	0.77^**^	0.56^**^	0.74^**^	0.71^**^	–																
TFI emotional	0.75^**^	0.79^**^	0.65^**^	0.58^**^	0.71^**^	0.57^**^	0.50^**^	0.69^**^	0.72^**^	–															
FTQ total	0.55^**^	0.57^**^	0.47^**^	0.46^**^	0.52^**^	0.39^**^	0.39^**^	0.51^**^	0.49^**^	0.54^**^	–														
TCS total	0.68^**^	0.66^**^	0.59^**^	0.61^**^	0.62^**^	0.52^**^	0.37^**^	0.63^**^	0.54^**^	0.63^**^	0.65^**^	–													
HQ total	0.57^**^	0.56^**^	0.38^**^	0.32^**^	0.56^**^	0.42^**^	0.43^**^	0.52^**^	0.59^**^	0.50^**^	0.31^**^	0.37^**^	–												
HQ attentional	0.40^**^	0.43^**^	0.27^**^	0.23^**^	0.47^**^	0.31^**^	0.28^**^	0.44^**^	0.44^**^	0.38^**^	0.22^**^	0.29^**^	0.87^**^	–											
HQ emotional	0.55^**^	0.55^**^	0.39^**^	0.33^**^	0.47^**^	0.37^**^	0.51^**^	0.44^**^	0.61^**^	0.46^**^	0.32^**^	0.33^**^	0.87^**^	0.61^**^	–										
HQ social	0.52^**^	0.49^**^	0.32^**^	0.29^**^	0.55^**^	0.42^**^	0.30^**^	0.48^**^	0.46^**^	0.47^**^	0.26^**^	0.36^**^	0.87^**^	0.70^**^	0.61^**^	–									
PSQ–30 total	0.47^**^	0.44^**^	0.23^**^	0.33^**^	0.48^**^	0.33^**^	0.25^**^	0.47^**^	0.43^**^	0.43^**^	0.26^**^	0.34^**^	0.49^**^	0.46^**^	0.32^**^	0.52^**^	–								
HADS anxiety	0.40^**^	0.35^**^	0.19^**^	0.22^**^	0.42^**^	0.30^**^	0.20^**^	0.40^**^	0.33^**^	0.39^**^	0.28^**^	0.39^**^	0.45^**^	0.40^**^	0.31^**^	0.48^**^	0.75^**^	–							
HADS depression	0.42^**^	0.40^**^	0.19^**^	0.29^**^	0.44^**^	0.29^**^	0.23^**^	0.39^**^	0.43^**^	0.39^**^	0.22^**^	0.27^**^	0.43^**^	0.37^**^	0.36^**^	0.41^**^	0.71^**^	0.65^**^	–						
WHOQoL physical health	–0.41^**^	–0.45^**^	–0.27^**^	–0.32^**^	–0.46^**^	–0.41^**^	–0.22^**^	–0.44^**^	–0.43^**^	–0.43^**^	–0.22^**^	–0.30^**^	–0.44^**^	–0.38^**^	–0.34^**^	–0.45^**^	–0.64^**^	–0.53^**^	–0.60^**^	–					
WHOQoL psychological	–0.37^**^	–0.34^**^	–0.16^*^	–0.24^**^	–0.41^**^	–0.26^**^	–0.13^*^	–0.37^**^	–0.33^**^	–0.35^**^	–0.25^**^	–0.30^**^	–0.37^**^	–0.35^**^	–0.26^**^	–0.38^**^	–0.70^**^	–0.68^**^	–0.71^**^	0.66^**^	–				
WHOQoL social relationships	–0.30^**^	–0.28^**^	–0.15^*^	–0.22^**^	–0.27^**^	–0.20^**^	–0.12	–0.32^**^	–0.30^**^	–0.29^**^	–0.12	–0.23^**^	–0.27^**^	–0.26^**^	–0.20^**^	–0.27^**^	–0.50^**^	–0.42^**^	–0.50^**^	0.46^**^	0.57^**^	–			
WHOQoL environment	–0.31^**^	–0.33^**^	–0.15^*^	–0.23^**^	–0.36^**^	–0.26^**^	–0.18^**^	–0.34^**^	–0.37^**^	–0.28^**^	–0.18^**^	–0.20^**^	–0.34^**^	–0.28^**^	–0.31^**^	–0.31^**^	–0.55^**^	–0.49^**^	–0.53^**^	0.64^**^	0.61^**^	0.43^**^	–		
WHOQoL overall quality of life	–0.38^**^	–0.38^**^	–0.23^**^	–0.25^**^	–0.39^**^	–0.35^**^	–0.16^**^	–0.40^**^	–0.39^**^	–0.41^**^	–0.24^**^	–0.28^**^	–0.41^**^	–0.39^**^	–0.34^**^	–0.35^**^	–0.63^**^	–0.57^**^	–0.60^**^	0.64^**^	0.65^**^	0.50^**^	0.61^**^	–	
WHOQoL overall health	–0.34^**^	–0.33^**^	–0.22^**^	–0.23^**^	–0.32^**^	–0.31^**^	–0.13^*^	–0.34^**^	–0.30^**^	–0.34^**^	–0.14^*^	–0.23^**^	–0.37^**^	–0.30^**^	–0.30^**^	–0.37^**^	–0.51^**^	–0.43^**^	–0.48^**^	0.72^**^	0.55^**^	0.37^**^	0.47^**^	0.56^**^	–

*THI, Tinnitus Handicap Inventory; TFI, Tinnitus Functional Index; FTQ, Fear of Tinnitus Questionnaire; TCS, Tinnitus Catastrophizing Scale; PSQ-30, Perceived Stress Questionnaire, HQ, Hyperacusis Questionnaire; HADS, Hospital Anxiety and Depression Scale; WHOQoL-BRE, World Health Organization Quality of Life Scale (short version)*.

** *Correlation is significant at the 0.01 level (2-tailed)*.

* *Correlation is significant at the 0.05 level (2-tailed)*.

#### Clinical validity

Subjects with a more severe clinical condition (permanent tinnitus, abrupt onset, and constant manifestation of tinnitus) tended to report more tinnitus-related impairments than subjects with a less severe clinical condition (occasional tinnitus, gradual onset, and intermittent manifestation of tinnitus). Table 6 summarizes the results of the Mann-Whitney *U*-tests.

**Table 6:** **Clinical validity of tinnitus-related questionnaires**.

***N* = 260**	**Tinnitus occurrence**			**Tinnitus onset**			**Tinnitus manifestation**		
**Questionnaire**	**Occasionally *n* = 34 med (Q1, Q3)**	**Permanently *n* = 226 med (Q1, Q3)**	***U*-test**	**Gradual *n* = 168 med (Q1, Q3)**	**Abrupt *n* = 92 med (Q1, Q3)**	**U-Test**	**Intermittent *n* = 68 med (Q1, Q3)**	**Constant *n* = 192 med (Q1, Q3)**	**U-test**
**THI total^b^**	**17.0 (6.0, 26.0)**	**26.0 (16.0, 42.0)**	2239.5^**^	**23.0 (12.0, 36.0)**	**28.0 (16.0, 46.0)**	**6691.0**	**20.0 (10.0, 31.5)**	**26.0 (16.0, 42.0)**	5256.0^*^
**TFI total^b^**	**11.0 (4.7, 25.4)**	**32.4 (16.3, 46.4)**	1948.0^**^	**29.2 (12.1, 42.6)**	**34.4 (14.0, 50.3)**	**6738.0**	**18.2 (9.8, 38.9)**	**32.6 (17.7, 46.7)**	4883.0^*^
Intrusive	20.0 (12.5, 37.5)	46.7 (26.7, 66.7)	1757.5^**^	43.3 (23.3, 63.3)	46.7 (23.3, 65.8)	7367.5	26.7 (14.2, 56.7)	46.7(26.7, 66.7)	4476.0^**^
Sense of control	11.7 (3.3, 40.8)	46.7 (20.0, 66.7)	2233.5^**^	40.0 (13.3, 63.3)	48.3 (24.2, 70.0)	6506.5^*^	36.7 (10.0, 62.5)	46.7 (20.0, 66.7)	5576.5
Cognitive	6.7 (0.0, 26.7)	20.0 (6.7, 43.3)	2708.0^*^	16.7 (6.7, 39.2)	23.3 (10.0, 55.8)	6671.0	10.0 (6.7, 30.0)	23.3 (6.7, 46.7)	5476.0^*^
Sleep	6.7 (0.0, 21.7)	16.7 (0.0, 44.2)	2970.5^*^	10.0 (0.0, 40.0)	16.7 (0.8, 50.0)	6741.0	8.3 (0.0, 39.2)	16.7 (0.0, 43.3)	5801.0
Auditory	10.0 (3.3, 36.7)	46.7 (22.5, 73.3)	1878.5^**^	46.7 (20.0, 66.7)	35.0 (10.8, 70.0)	7214.0	30.0 (10.0, 55.8)	50.0 (20.0, 73.3)	4746.5^**^
Relaxation	10.0 (0.0, 23.3)	26.7 (10.0, 53.3)	2426.0^**^	20.0 (4.2, 46.7)	33.3 (10.0, 60.0)	6370.5^*^	18.3 (3.3, 32.5)	26.7 (10.0, 56.7)	5278.0^*^
Quality of life	2.5 (0.0, 20.6)	22.5 (5.0, 47.5)	2197.0^**^	20.0 (2.5, 42.5)	22.5 (3.1, 47.5)	7245.0	11.3 (2.5, 26.9)	22.5 (5.0, 47.5)	5205.0^*^
Emotional	1.7 (0.0, 4.2)	6.7 (0.0, 16.7)	2415.5^**^	6.7 (0.0, 13.3)	6.7 (0.8, 26.7)	6412.0^*^	3.3 (0.0, 10.0)	6.7 (0.0, 20.0)	5314.5^*^
**FTQ total^b^**	**4.0 (2.8, 4.0)**	**4.0 (3.0, 6.0)**	2921.0^*^	**4.0 (3.0, 6.0)**	**4.0 (3.0, 6.0)**	**7036.0**	**4.0 (3.0, 6.0)**	**4.0 (3.0, 6.0)**	**5913.5**
**TCS total^b^**	**8.0 (2.0, 16.0)**	**12.0 (6.0, 21.0)**	2860.5^*^	**11.0 (6.0, 19.0)**	**13.5 (5.0, 22.0)**	**7184.5**	**10.0 (4.0, 16.0)**	**12.5 (6.0, 21.0)**	5344.5^*^
**PSQ-30 total^b^**	**0.2 (0.2,0.4)**	**0.3 (0.2,0.4)**	**3735.0**	**0.3 (0.2,0.4)**	**0.3 (0.1,0.4)**	**6874.0**	**0.3 (0.2,0.4)**	**0.3 (0.2,0.4)**	**6370.5**
**HQ total^b^**	**14.5 (8.0, 22.5)**	**17.0 (10.0, 25.0)**	**3422.0**	**15.0 (8.3, 24.0)**	**20.0 (12.0, 27.8)**	6204.5^*^	**15.0 (9.3, 21.8)**	**17.5 (10.0, 26.0)**	**5681.5**
Attentional	4.5 (2.8, 7.0)	5.0 (2.0, 8.0)	3817.0	4.0 (2.0, 6.8)	6.0 (3.0, 8.0)	6154.5^*^	4.0 (2.0, 6.0)	5.0 (2.0, 8.0)	5458.5^*^
Emotional	5.0 (3.0, 9.3)	7.0 (4.0, 11.0)	3084.0	6.0 (4.0, 10.0)	8.0 (5.0, 12.0)	6522.0^*^	6.0 (4.0, 10.0)	7.0 (4.0, 11.8)	5738.5
Social	5.0 (2.0, 7.5)	5.0 (2.0, 8.0)	3641.5	4.0 (2.0, 7.8)	6.0 (3.0, 9.0)	6409.5^*^	5.0 (2.0, 7.0)	5.0 (2.0, 8.8)	6204.5
**HADS^b^**									
Anxiety	4.0 (2.0, 8.0)	4.0 (2.0, 8.0)	3766.0	4.0 (2.0, 7.0)	5.0 (2.0, 8.0)	6486.5^*^	4.0 (2.0, 7.0)	5.0 (2.0, 8.0)	6085.5
Depression	1.0 (1.0, 4.0)	2.0 (1.0, 5.0)	3223.5	2.0 (1.0, 4.0)	3.0 (1.0, 6.8)	6501.0^*^	2.0 (1.0, 5.0)	2.0 (1.0, 5.0)	6475.5
**WHOQoL-BREF**^a^									
Physical health	17.1 (14.1, 18.3)	15.4 (13.7, 17.7)	3284.5	16.6 (13.7, 17.7)	14.9 (13.1, 17.1)	6326.5^*^	16.0 (13.3, 17.7)	15.4 (13.7, 17.7)	6277.0
Psychological	16.0 (14.0, 17.3)	16.0 (14.0, 17.3)	3734.5	16.0 (14.7, 17.3)	15.3 (13.3, 16.7)	6169.5^*^	16.0 (13.5, 17.3)	16.0 (14.0, 17.3)	6468.5
Social relationships	14.7 (12.0, 16.0)	14.7 (13.3, 16.0)	3820.0	14.7 (13.3, 16.0)	14.7 (12.3, 16.0)	7113.5	14.7 (12.0, 16.0)	14.7 (13.3, 16.0)	6034.5
Environment	16.5 (15.5, 17.1)	16.5 (15.0, 18.0)	3834.5	16.5 (15.1, 18.0)	16.0 (15.0, 17.5)	7009.0	16.5 (15.0, 18.0)	16.5 (15.0, 18.0)	6247.0
Overall quality of life	4.0 (4.0, 5.0)	4.0 (4.0, 5.0)	3638.0	4.0 (4.0, 5.0)	4.0 (3.0, 5.0)	5965.0^*^	4.0 (4.0, 5.0)	4.0 (4.0, 5.0)	6338.5
Overall health	4.0 (3.0, 4.0)	4.0 (3.0, 4.0)	3763.5	4.0 (3.0, 4.0)	3.0 (2.0, 4.0)	6606.5^*^	4.0 (3.0, 4.0)	4.0 (3.0, 4.0)	6436.0

*THI, Tinnitus Handicap Inventory; TFI, Tinnitus Functional Index; FTQ, Fear of Tinnitus Questionnaire; TCS, Tinnitus Catastrophizing Scale; PSQ-30, Perceived Stress Questionnaire; HQ, Hyperacusis Questionnaire; HADS, Hospital Anxiety and Depression Scale; WHOQoL-BREF, World Health Organization Quality of Life Scale (short version)*.

** *Correlation is significant at the 0.001 level*.

* *Correlation is significant at the 0.05 level*.

a *Higher score, higher quality of life*.

b *Higher score, higher impairment*.

*Summary of Mann-Whitney U-Tests in relation to occurrence (occasional vs. permanent), onset (gradual vs. abrupt), and manifestation (intermittent vs. constant)*.

## Discussion

Overall, the Swedish versions of the tinnitus-specific questionnaires showed good internal consistency, test–retest reliability, construct, as well as clinical validity. Internal consistency was excellent (α > 0.90) for the THI, TFI, TCS, HQ, and PSQ-30, good for the HADS (0.80 ≤ α ≤ 0.90), and acceptable for the FTQ (0.70 ≤ α ≤ 0.80). However, the subscale “social relationships” of the WHOQoL-BREF showed low internal consistency that fell short of reaching the conventional cut-off value of α ≤ 0.70.

Test–retest reliability was acceptable, except for the subscale sense of control of the TFI (ICC = 0.68) and for 15 items of the TSCHQ that includes descriptive data about tinnitus. The comparison of TSCHQ scores with previously published descriptive analyses by the Tinnitus Research Initiative (Schecklmann et al., 2014) showed very similar prevalence for specific items. For instance, gradual perception of tinnitus at its onset was reported by 64.6% of subjects in STOP vs. 50% in the TRI. Similarly, high-frequency perceptions were reported by 65.8% of subjects in STOP vs. 72% in the TRI. 73.8% of subjects in the STOP reported constant tinnitus in comparison to 84% in the TRI. Cohen's kappa coefficients of several TSCHQ items (e.g., first tinnitus experience, manifestation of tinnitus over time, or suffering from headaches) were below the cut-off value of *k* > 0.60. However, this result does not mean that the questionnaire is not reliable *per-se*. The low kappa values may reflect (a) variables that differ in time or are fluctuating, (b) variables that are not accurately remembered, (c) that subjects did not understand the item, and (d) how reliably or conscientiously subjects respond to questionnaires. Nonetheless, this finding suggests that caution should be taken in the interpretation of some items of the TSCHQ. The test–retest reliability of the TSCHQ should also be investigated in a different sample in order to find out whether the phrasing of specific questions should be modified—this could also apply to the original English version of the TSCHQ.

Backward translation is often conducted to ensure the reliability of the forward translation, however, we found no guideline on how to score the reliability of a backward translation. We considered one-word change in the translated version, as a meaningful difference when comparing to the original version. Using this criterion, we found that in the case of English-Swedish translations, near 60% of backward-translated items from the TSCHQ and the TFI differed from the original version. With shorter sentences, as those found in the PSQ-30, this number went down to 40%. Importantly, of all backward-translated items in which a change from the original version was observed, only 6% of them had potentially altered meaning. Verification of the Swedish items helped confirming that they were culturally adapted and thus appropriate for testing. The low score for the subscale sense of control of the TFI (ICC = 0.68) could potentially derive from translation failures. The verb “*to cope*” in English has the equivalent “*att hantera*” in Swedish, but which has additional meanings such as “*to handle*” or “*to manage*.” Such differences, when evaluating the “sense of control” could alter the test–retest reliability. Potentially, this variability in the test–retest sessions might not necessarily occur in a more severe group of individuals such as those recruited in clinics, which is not the case with the STOP cohort (population based).

Indeed, it is possible that the low values obtained for some of the items of the TSCHQ are due to the fact that the population tested within the STOP includes participants from the general population and not clinical (outpatient) individuals. When comparing the scores of STOP participants with the scores obtained in other studies, we observed that the scores of the different questionnaires were lower than normal. Because most studies failed to report median values, we compared the mean values. Our average THI score was 28.34 in comparison to the range of 40–55 found in the literature (Kaldo et al., 2007; Westin et al., 2011; Albu and Chirtes, 2014; Jasper et al., 2014). The TFI average was 31.74 vs. 40.6 (Fackrell et al., 2016), so that the overall score was lower than that typically found in the literature. Similarly, the anxiety level of 5.12 measured with the HADS was lower than that reported in other studies (6.2–8.7; Kaldo et al., 2007; Westin et al., 2011; Albu and Chirtes, 2014; Jasper et al., 2014). The average of 3.44 for depression in the STOP cohort was also lower than the range of 4.05–6.5 described in the literature (Kaldo et al., 2007; Westin et al., 2011; Albu and Chirtes, 2014; Jasper et al., 2014). The average values of 13.86 of tinnitus-specific cognitions measured with the TCS were almost two times less than the baseline score of 21.11 in the study by Cima et al. (2012). Fear-reactivity as measured by the FTQ was 4.57 in the STOP cohort vs. 7.25 (Cima et al., 2012) at baseline. This finding may be due to the fact that all patients in the Cima trial had severely irritating tinnitus at baseline with an average THI score of 38.96 (*SD* 22.88; Cima et al., 2012) in contrast to our study sample who had significantly less severe tinnitus with an average score of 28.34. The STOP values were more comparable with the values for the 12 month follow-up trial by Cima et al. (2012) that were 11.73 for the TCS and 4.20 for the FTQ. Our study participants only seemed to be mildly affected by tinnitus compared to the RCT population. The average scores for quality of life were very similar to those reported in the literature (Abbott et al., 2009; Kreuzer et al., 2014; Schecklmann et al., 2014). Most published studies involved patients with tinnitus recruited in clinical centers or from medical registries, whereas the subjects recruited in the initial phase of the STOP were representative of the general population that may include individuals diagnosed and not diagnosed with tinnitus. As a consequence, this difference may potentially result in lower severity scores for all questionnaires. These findings emphasize the need of testing these questionnaires in a group of outpatients from clinics in Sweden.

Interestingly, the hyperacusis scores of the HQ were very similar to those found in the literature (Fackrell et al., 2015). However, using the criterion of >28 of Khalfa et al. (2002), we would obtain a proportion of 17.7% of subjects with hyperacusis, but this percentage is well below the reported 40–55% typically found in the tinnitus population (Baguley, 2003; Schecklmann et al., 2014). Indeed, reevaluation of the cut-off threshold has recently been recommended (Fackrell et al., 2015).

The potential to distribute questionnaires online has large benefits over paper versions, both in research and in clinical settings, because large data sets can be created with minimal administrative efforts. Moreover, the use of online questionnaires may precede anamnesis and audiological assessment to allow a more focused discussion at the clinic. Distributing the HADS and HQ questionnaires over the internet has proved successful and validated against pen and paper (Andersson et al., 2002, 2003; Thorén et al., 2012). The internal consistency and reliability of the online questionnaires tested here suggests that they could be used in paper versions in clinics that do not yet have the IT infrastructure to implement web-based versions.

## Conclusions

This study shows the likely suitability of the Swedish versions of the THI, the TFI, the TCS, the FTQ, the HQ, the PSQ-30, the HADS, and the WHOQoL-BREF for measuring outcome in a clinical and research setting. The reliability and validity of these questionnaires translated into Swedish are comparable with that of the original English language versions. Some items of the TSCHQ may have to be removed or rewritten to further improve the reliability of this questionnaire. Additional research may be required to evaluate the sensitivity of each questionnaire in longitudinal studies and their usefulness for measuring treatment outcomes.

## Author contributions

BL, WS, BC, RC, and CC designed the study. EI, RH, VP, CL, NE, and CC provided a consensus agreement on the final translated questionnaires. NE and CC developed the web-survey, coordinated the recruitment of subjects, and collected the data. KM and CC analyzed the data. CC, WS, and KM drafted the initial version of the manuscript. All authors contributed to the final version of the manuscript.

### Conflict of interest statement

The authors declare that the research was conducted in the absence of any commercial or financial relationships that could be construed as a potential conflict of interest.
